# Predictive Value of Neutrophils, Lymphocytes, Platelets, Neutrophil–Lymphocyte Ratio (NLR), and Platelets–Lymphocyte Ratio (PLR) in Preterm Labor: Single and Combined Indices

**DOI:** 10.1155/ogi/5401809

**Published:** 2026-06-12

**Authors:** Emad Elmaradny, Saud Aman, Mohammed Alaskar, Reem Alnefayi, Ali Almuhesseny, Hashem Alshreef, Malak Alshammari, Adnan Alhazmi, Lulwah Altraifi, Amal AlSaedi

**Affiliations:** ^1^ Department of Obstetrics and Gynecology, Al Yamamah Hospital, Riyadh, Saudi Arabia

**Keywords:** lymphocytes, neutrophil–lymphocyte ratio (NLR), neutrophils, platelets, platelet–lymphocyte ratio (PLR), preterm birth, preterm labor

## Abstract

**Introduction:**

Maternal neutrophils (N), lymphocyte, platelets (PLTs), neutrophil–lymphocyte ratio (NLR), and platelets–lymphocyte ratio (PLR) have been used to predict preterm birth. We studied changes in these markers in preterm labor (PTL) and whether they can serve as single or combined predictive indicators.

**Material and Methods:**

Patients in premature labor participated in a retrospective case–control study. CBC, Hb, HCT, lymphocytes, N, PLTs, NLR, and PLR were measured and compared between total preterm cases (20 0/7–36 6/7 weeks) and normal pregnancies. Subgroups of early preterm labor (EPL) (20 0/7–33 6/7 weeks) and late preterm labor (LPL) (34 0/7–36 6/7 weeks) were also compared with gestational‐age‐matched controls. ROC curves were utilized to estimate the cutoff, specificity, sensitivity, and area under the curve (AUC) for every marker. According to cutoff levels of each marker, a score system was created (0–4). Combined Score Index and Systemic Immune‐Inflammatory Index (SII) were calculated for total, early, and late preterm groups.

**Results:**

A total of 1000 patients with PTL were included. No significant difference was found in lymphocytes between groups. N, PLTs, NLR, and PLR were significantly higher in total PTL vs. controls. In EPL, N, PLTs, NLR, and PLR were significantly elevated (*p* ≤ 0.0001, 0.0001, 0.0002, and 0.0009). Similarly, LPL showed significant differences (*p* ≤ 0.0003, 0.01, 0.0008, and 0.05). These markers were also significantly higher in EPL vs. LPL (*p* ≤ 0.0001, 0.05, 0.0001, and 0.05). AUC values in early preterm for N, PLTs, NLR, and PLR were 0.640, 0.626, 0.627, and 0.593, respectively, and in late preterm, 0.611, 0.568, 0.610, and 0.581—indicating a weak predictive value. A high score index (3‐4) was more frequent in total (57%), early (31.7%), and late (50.27%) preterm compared with controls (38.5%, 15.56%, and 31.86%). AUC of Combined Score Index was 0.604, 0.630, and 0.611 and for SII, 0.660, 0.684, and 0.622 in total, early, and late preterm, respectively, showing statistically significant but weak predictive values.

**Conclusion:**

Significant changes in N, PLTs, NLR, and PLR (both as individual and combined markers) are observed in PTL groups, but their predictive value for prematurity remains limited.

**KeyMessage**
• Although neutrophils, platelets, NLR, and PLT are increased in preterm labor, they are not highly predictive for preterm labor.

## 1. Introduction

A preterm birth occurs when the baby is born between 20 0/7 and 36 6/7 weeks of pregnancy [1]. Preterm delivery before 37 weeks of pregnancy have passed is a very critical obstetric problem and a major cause of perinatal complications. It affects 5%–18% of all pregnancies. Roughly, 70%–80% of preterm births are the outcome of the spontaneous onset of labor [2]. Worldwide, about 15 million babies are delivered preterm every year. Approximately 75% of perinatal deaths are caused by preterm labor (PTL), and almost 1.1 million babies expire yearly due to their prematurity [3].

Preterm birth is classified based on gestational age at delivery. Births occurring between 34 weeks and 36 weeks and 6 days are classified as late preterm births, whereas those between 32 weeks and 33 weeks and 6 days are referred to as moderate preterm births, very preterm (28 to less than 31 weeks and 6 days), and extremely preterm (less than 28 weeks) [1].

The risks of morbidity and mortality are greater between babies delivered before 32 weeks of gestation. Even the survivor’s offspring will face both short‐ and long‐term sequelae [4].

During pregnancy, there are considerable maternal hematological changes throughout the pregnancy. In the first and second trimesters of pregnancy, the leukocyte count decreases and then increases in the third trimester [5]. A significant decrease in platelet (PLT) count has been seen since the 32nd week of gestation [6].

PTL has multiple etiologies, involving intrauterine infection, inflammation, genetic factors, endocrine disorders, uteroplacental ischemia, cervical incompetence, hemorrhage, early activation of the fetal endocrine system, and immunological factors. Other risk factors involved in PTL include hypertensive diseases during pregnancy, multiple pregnancies, polyhydramnios, and uterine anomalies [7].

Modern clinical assessments for the detection of preterm delivery, including cervical length measurement by transvaginal sonography, fetal fibronectin assays, and bacterial vaginosis, have not been effectively adjusted for the identification of preterm delivery. In addition, these measures are expensive and time‐consuming. Moreover, some studies have revealed the low predictive accuracy of these tests. Hence, there is still a requirement for further markers for predicting preterm birth [8].

Although the triggering factor for PTL has not been exactly identified, the fundamental role of systemic and subclinical infections is well recognized in several studies [9, 10]. Inflammation has been linked to the processes that initiate and maintain both term and PTL. It has been proposed that inflammatory and infectious conditions increase the hazard of premature labor. Nevertheless, of these causes, inflammation is the main pathologic process of PTL and PPROM [11, 12].

Clinical and subclinical infection and inflammation are the main contributing factors to the pathogenesis of preterm birth. Inflammation, clinically manifested by pain, redness, swelling, and fever, initiates the effect of inflammatory mediators on local blood vessels and tissues. In contrast, subclinical inflammation is recognized by the infiltration of neutrophils (N), macrophages, and lymphocytes within tissues. Systemic inflammation changes the absolute and relative counts of N and lymphocytes. An increase in these cells is also symptomatic of subclinical infection [14].

Neutrophil–lymphocyte ratio (NLR) or platelet–lymphocyte ratio (PLR) has also been identified as prospective inflammatory markers in PTL. It is thought that simple measurements of the NLR and PLR are excellent predictors of inflammation [15, 16].

Clinically, a complete blood count (CBC) is a simple, affordable, and easily accessible laboratory test. Prior studies have determined a correlation between the increased in PLT counts and infection, inflammation, and malignancy [17].

According to recent data, determining the ratio of blood cell subtypes, such as the NLR, the lymphocyte to monocyte ratio (LMR), and the PLR, may have important significance for diseases related to chronic or subclinical inflammation [18]. Investigations have shown that using a single biomarker to predict TPL is still challenging. It is believed that there may be variations in the parameters of maternal serum in PTL, PPROM, and threatened preterm [15].

Recently, it has been reported that serum maternal CBC markers can be used more precisely to predict the occurrence of preterm birth compared with other conventional inflammatory indicators. Using a single inflammatory biomarker to predict PTL is still difficult [19]. Therefore, there is a need for additional markers predicting preterm birth among patients with spontaneous PTL. Many studies reported an increase in N counts, a decrease in lymphocyte counts, and an increase in NLR and PLR that may predict PTL [15–20].

It was suggested that combination of N, PLTs, NLR, and PLR can increase the accuracy and sensitivity of predicting outcomes compared with using them individually [21].

Many studies tried to create a combined system or score by adding some of the risk factors together and evaluating its clinical significance in the prediction of PTL. Combined Score Index is a simplified score developed based on calculation of cutoff level of each factor by receiver operating characteristic (ROC) curve. It was used to assess the predictive value of combined PLT count, NLR, and PLR in preeclampsia [22].

System Immune Inflammation Index (SII) is a marker demonstrating both inflammatory and immune responses. High SII levels imply the severity of inflammation and have been related with poor prognosis in conditions such as cancer, cardiovascular disease, and infection. Kırat S showed a significantly higher SII in PTL [23].

Sert ZS reported that SII was more valued than NLR and PLR in the early detection of PTL. SII can help recognize pregnant women at risk of developing PTL [24].

The aim of this research was to assess the changes in inflammatory parameters in PTL and whether they could be used as clinical predictive markers for PTL as separate inflammatory markers or in combination.

## 2. Material and Methods

This study was conducted retrospectively in the Obstetrics and Gynecology Department of AL‐Yamamah Hospital, Riyad, Saudi Arabia. The Ethical Committee of Riyadh Second Health Cluster approved the study, and the Institutional Review Board (IRB log no. 24–231E) was obtained. Personal data such as name address phone number and ID were blind to researchers. Written informed consent was obtained from all the patients involved.

### 2.1. Study Participants

All patients admitted due to spontaneous PTL with intact membranes between 20 0/7 and 36 6/7 weeks from January 2020 to December 2022 were collected.

### 2.2. Exclusion Criteria

To avoid physiological labor–related inflammation rather than true predictive markers, patients with imminent PTL, cervical dilatation, or delivered within 48 h of hospital admissions were not involved in this study.

Patients with PPROM, significant obstetric or medical complications, and nonreassuring maternal and fetal status information were excluded. Patients with evidence of any infection and a history of cervical insufficiency and premature birth in a previous pregnancy were excluded from the study.

Clinical chorioamnionitis, placental abruption, antepartum hemorrhage, twin, or multiple pregnancies, and intrauterine growth restriction were not included in the study.

Patients with pregnancy‐induced hypertension, preeclampsia, gestational diabetes mellitus, recurrent abortion history, cardiopulmonary diseases, thyroid disease, rheumatic metabolic diseases, hematological diseases, deep venous thrombosis, tuberculosis, and urinary system infections were excluded.

In this study, patients in PTL are further categorized into 2 subgroups, late preterm labor (LPL) (34–36 6/7 weeks) and early preterm labor (EPL) (20 0/7–33 6/7 weeks).

A control group of patients who had a normal pregnancy during antenatal care with comparable maternal age, parity, and delivered normally at term were also collected randomly (early control between 20 0/7 and 33 6/7 weeks and late control between 34 and 36 6/7 weeks). CBCs obtained from the computerized data in Al Yamamah Hospital during routine antenatal care at the same gestational week (20 0/7–36 6/7 weeks) were used.

The gestational age is calculated using the sure last menstrual period and ultrasonographic screening in the first trimester. Demographic data from patient files and laboratory parameters were recorded.

Blood sample was taken at the time of admission. CBC, Hb, HCT, N, lymphocytes, PLTs, NLR, and PLR were collected from patients’ files. NLR and PLR values were calculated by dividing the absolute N and PLT counts, respectively, by the absolute lymphocyte counts.

### 2.3. Statistical Methods

CBC parameters among the six groups (total preterm, EPL, LPL, total control normal pregnancy, early pregnancy, and normal late pregnancy) were compared, and the association of gestational age at delivery with CBC parameters was evaluated. The data collected were analyzed using SPSS (Version 22.0, SPSS Inc., Chicago, IL, USA). Descriptive statistics were presented as the mean ± standard deviation of normal distributed numerical data. Students’ *t*‐test for continuous variables and chi‐square statistics for categorical variables were used to investigate the differences between the groups.

### 2.4. ROC Graphs

ROC analysis was applied to assess the specificity and sensitivity of each marker. You den index was used to determine the best cutoff point in ROC analysis. We calculated the area under the curve (AUC) and the 95% confidence intervals of this area.

The optimal cutoff value resulting in the highest sum of sensitivity and specificity for each marker was determined.

The optimal cutoff values for N, PLTs, NLR, and PLR were determined using the Youden Index (Ј). For each parameter, the index was calculated using the formula: (Ј = Sensitivity + Specificity−1).

A *p* value < 0 0.05 was considered statistically significant for all analyses. In the analysis of AUC, the clinical significance of determining the risk group was evaluated as 0.9–1: excellent, 0.8–0.9: good, 0.7–0.8: moderate, 0.6–0.7: weak, and 0.5–0.6: bad or failure for AUC.

### 2.5. Combined Score Index

For each cutoff level, each patient may or may not reach the cutoff level. This will create a binary system 0 or 1 for each cutoff level of the three parameters. A combination of N, PLTs, NLR, and PLR cutoff levels for each patient will create a scoring system ranging between 0, 1, 2, 3, and 4. Score 0 (*very low or no risk*) means none of the cutoff levels of N, PLTs, NLR, and PLR were reached. In Score 1 (*low risk*), only one cutoff level was achieved. Score 2 (*mild risk*), Score 3 (*moderate risk*), and Score 4 (*high risk*) indicated 2, 3, and 4 cutoff levels were reached. ROC curve of these combined score indices in total, early (20 0/7 33 6/7 w), and late (34 36 6/7 w) preterm were calculated compared with normal pregnancy.

SII was defined as [neutrophil count × platelet count/lymphocyte count]. Optimal thresholds were newly calculated for our cohort using ROC curve analysis. The cutoff points were determined by the maximum Youden Index.

## 3. Results

Patients with spontaneous PTL between 20 0/7 and 36 6/7 weeks were involved in this study after exclusion criteria resulting in 1000 patients, 470 with EPL and 530 with LPL. Control cases included 200 patients with normal pregnancies who delivered after 37 weeks. Data were collected from 90 control cases during antenatal care (20 0/7–33 6/7 w) and from 110 cases (34–36 6/7 w).

CBC data were collected from medical records obtained from preterm cases or during antenatal care in control cases.

Demographic data and various parameters (mean ± SD, CI, and mean difference) analyzed in total preterm cases and total normal pregnancies are shown in Table 1. No significant differences were found in maternal age, parity, G.A, Hb, HCT, and lymphocytes in total PTL cases and total normal pregnancy women.

**TABLE 1:** Maternal age, parity, gestational age, Hb, HCT, Lymphocytes, platelets, NLR and PLR in preterm labor (20 0/7–36 6/7 weeks) and normal pregnancy.

	**Preterm labor (20+0–36 6/7 w) *N* = 1000**	**CI**	**Normal pregnancy (20+0–36 6/7 w) *N* = 200**	**CI**	**Mean difference MD**	**p** **value ≤**

Maternal Age (Y)	29.91 ± 6.25	29.51–30.29	29.87 + 5.86	29.00–30.65	−0.04	0.9
Parity	2.25 ± 2.26	2.11–2.39	2.21 ± 2.36	1.88–2.53	−0.04	0.8
GA (in weeks)	33.37 ± 3.28	33.17–33.57	33.30 ± 3.37	32.83–33.77	−0.07	0.558
Hb (g/dL)	10.52 ± 1.78	10.41–10.64	10.36 ± 1.87	10.11–10.63	−0.16	0.2
HCT (%)	32.56 ± 4.92	32.28–32.86	33.00 ± 6.58	32.11–33.94	0.44	0.2
Lymphocytes (10 [9]/L)	2.41 ± 0.74	2.37–2.46	2.50 ± 0.95	2.18–2.45	0.09	0.127
Neutrophils (10 [9]/L)	9.12 ± 3.18	8.92–9.32	7.70 ± 2.59	7.34–8.07	−1.42	0.0001
Platelets (PLT) (10 [9]/L)	257.38 ± 71.13	252.98–261.81	231.88 ± 60.15	223.63–240.41	−25.5	0.0001
NLR	4.19 ± 1.89	3.99–4.23	3.38 ± 1.53	3.16–3.59	−0.81	0.0001
PLR	115.49 ± 45.23	112.68–118.29	102.50 ± 42.37	96.59–108.40	−12.99	0.0002

Also, no significant differences were found in maternal age, parity, G.A, Hb, HCT, and lymphocytes in early or late PTL and control groups.

N, PLT, NLR, and PLR were significantly higher in total PTL compared with total normal pregnancy (9.12 ± 3.18, 7.70 ± 2.59, 257.38 ± 71.13, 231.88 ± 60.15, 4.19 ± 1.89, 3.38 ± 1.53, 115.49 ± 45.23, and 102.50 ± 42.37, respectively) (Table 1).

There was also a significantly higher difference between N, PLTs, NLR, and PLR in EPL (20 0/7–33 6/7 w) compared with normal healthy pregnant women (*p* ≤ 0.0001, 0.0001, 0.0002, and 0.0009, respectively).

Also, a significant higher difference was also detected between LPL (34–36 6/7 w) and control normal pregnancy cases (*p* ≤ 0.0003, 0.01, 0.0008, and 0.05, respectively) (Table 2).

**TABLE 2:** Maternal age, parity, gestational age, Hb, HCT, Lymphocytes, platelets, NLR and PLR in early (20 0/7–33 6/7w) and late preterm labor (34–36 6/7 w) and normal early and late pregnancy.

	**Early preterm labor (20 0/7–33 6/7w) N = 470**	**Late preterm labor (34–36 6/7 w) N = 530**	**p** **value early vs late preterm**	**Early normal pregnancy (20 0/7–336/7 w) N = 90**	**Late normal pregnancy (34–36 6/7 w) N = 110**	**P** **value early vs late Normal pregnancy**	**p** **value early preterm vs early normal pregnancy (20 0/7–33 6/7w)**	**p** **value late preterm labor vs late Normal pregnancy (34–36 6/7 w)**

Maternal Age (Y)	29.56 ± 6.19	30.22 ± 6.29	*p* ≤ 0.09	29.54 ± 5.80	30.15 ± 5.87	*p* ≤ 0.47	*p* ≤ 0.98	*p* ≤ 0.91
Parity	2.07 ± 2.30	2.40 ± 2.22	*p* ≤ 0.02	2.03 ± 2.29	2.36 ± 2.40	*p* ≤ 0.32	*p* ≤ 0.89	*p* ≤ 0.88
GA (in weeks)	30.78 ± 3.16	35.67 ± 0.47	*p* ≤ 0.0001	30.49 ± 3.26	35.61 ± 0.49	*p* ≤ 0.0001	*p* ≤ 0.44	*p* ≤ 0.21
Hb (g/dL)	10.62 ± 1.75	10.44 ± 1.81	*p* ≤ 0.12	10.37 ± 1.80	10.35 ± 1.92	*p* ≤ 0.92	*p* ≤ 0.23	*p* ≤ 0.62
HCT (%)	32.76 ± 4.89	32.38 ± 4.93	*p* ≤ 0.23	32.78 ± 4.45	33.18 ± 7.90	*p* ≤ 0.67	*p* ≤ 0.96	*p* ≤ 0.17
Lymphocytes (10 [3]/μL)	2.41 ± 0.76	2.41 ± 0.72	*p* ≤ 0.98	2.42 ± 0.81	2.54 ± 1.05	*p* ≤ 0.38	*p* ≤ 0.94	*p* ≤ 0.12
Neutrophils	9.75 ± 3.18	8.56 ± 3.09	*p* ≤ 0.0001	8.05 ± 2.56	7.41 ± 2.58	*p* ≤ 0.08	*p* ≤ 0.0001	*p* ≤ 0.0003
Platelets (PLT)	261.85 ± 71.94	253.45 ± 70.18	*p* ≤ 0.05	228.23 ± 54.35	234.83 ± 64.32	*p* ≤ 0.44	*p* ≤ 0.0001	*p* ≤ 0.01
NLR	4.41 ± 2.02	3.84 ± 1.72	*p* ≤ 0.0001	3.56 ± 1.54	3.24 ± 1.52	*p* ≤ 0.15	*p* ≤ 0.0002	*p* ≤ 0.0008
PLR	118.38 ± 48.51	112.92 ± 41.95	*p* ≤ 0.05	100.58 ± 34.65	104.18 ± 47.65	*p* ≤ 0.55	*p* ≤ 0.0009	*p* ≤ 0.05

*Note:* Data are shown in Mean ± SD.

In this study, N, PLT, NLR, and PLR in EPL (20 0/7–33 6/7 w) were significantly higher compared with LPL (34–36 6/7 w) (*p* ≤ 0.0001, 0.05, 0.0001, and 0.05, respectively).

### 3.1. ROC Curve and Predictive Values

**FIGURE 1:**
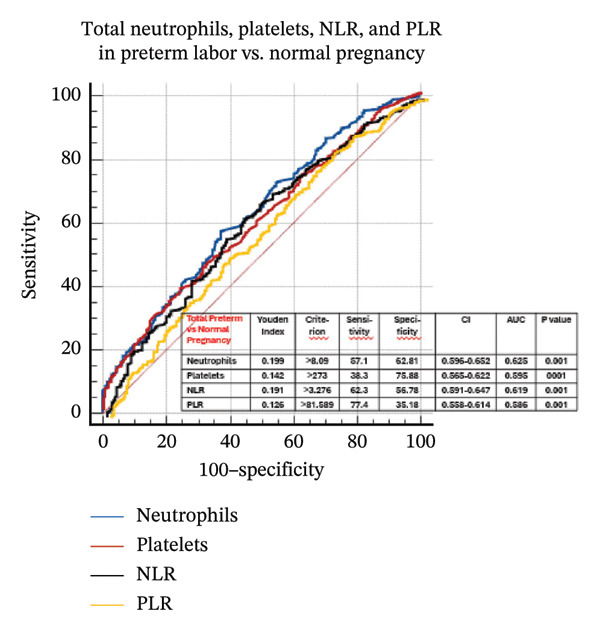
Roc curve of neutrophils, platelets, NLR and PLR in total preterm compared to normal pregnancy.

In total preterm cases, ROC curve had AUC of N, PLTs, NLR, and PLR were higher compared with total normal pregnancy women (0.625, 0.595, 0.615, and 0.586) (Figure 1).

**FIGURE 2:**
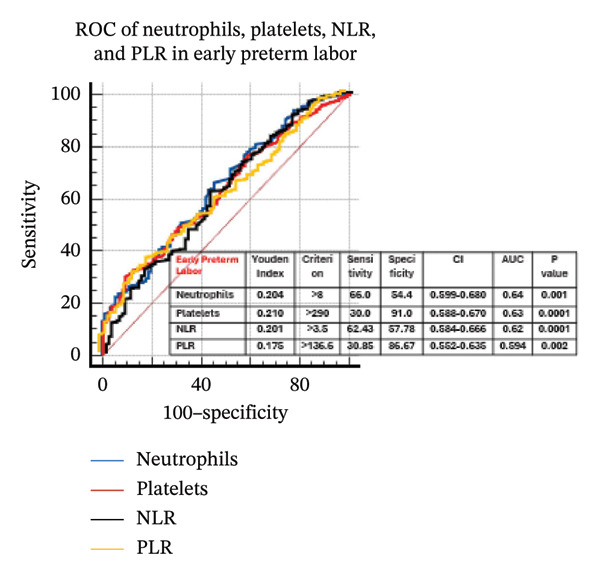
ROC curve of neutrophils, platelets, NLR and PLR in Early (20 0/7–33 6/7 w).

The AUC of ROC curves for N, PLTs, NLR, and PLR in EPL (20 0/7–33 6/7 w) were also higher (0.64, 0.63, 0.625, and 0.594) compared with normal pregnancy (*p* < 0.001, 0.001, 0.001, and 0.02, respectively) (Figure 2). Although ROC curve analysis showed that N, PLTs, NLR, and PLR in EPL (20 0/7–33 6/7 w) had a high AUC, they were clinically weak as single markers in predicting early preterm delivery (0.64, 0.63, 0.62, and 0.594).

**FIGURE 3:**
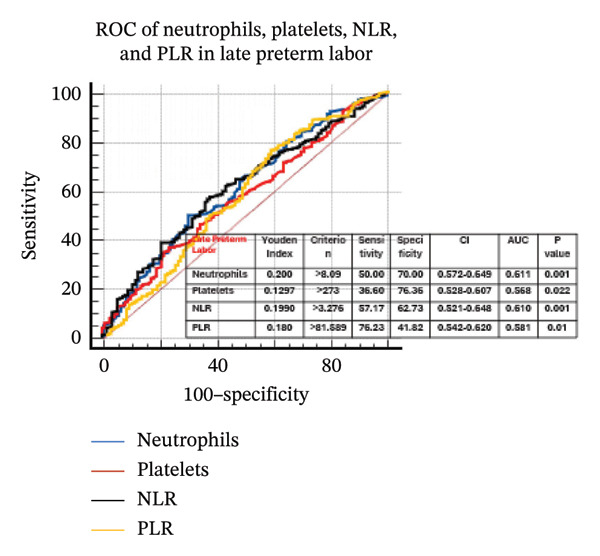
ROC curve of neutrophils, platelets, NLR and PLR in Late (34–36 6/7 w).

AUC in LPL (34–36 6/7 w) were higher compared with normal pregnancy (0.611, 0.568, 0.610, and 0.581) (Figure 3). Also, AUC of N, PLTs, NLR, and PLR in LPL (34–36 6/7 w) had a weak clinical predicting value for LPL as single markers.

Total data on AUC, p values, criterion (cutoff levels), sensitivity, and specificity are shown in Figures 2 and 3.

## 4. Combined Score Indices

The percentage (%) of patients reached cutoff levels for N, PLTs, NLR, and PLR (scores 4,3,2,1, and 0) in total preterm and early (20 0/7–33 6/7 w) and late (34–36 6/7 w) PTL and in normal pregnancy were calculated. High scores (4 + 3) and low scores are shown in Table 3.

**TABLE 3:** Combined score index of high (4 + 3) and low (1 + 0) scores of Total (20 0/7–36 6/7 w), early (20 0/7–33 6/7 w) and Late (34 0/7–36 6/7 w) preterm labor vs normal pregnancy.

	**CasesNo.**	**Score 4%**	**Score 3%**	**Score 2%**	**Score 1%**	**Score 0%**	**High score (4 + 3) %**	**Low score (0 + 1) %**

Total Preterm	1000	38.6	18.4	5.6	15	22.4	57	37.4
Total Normal Pregnancy	200	24.5	14	6.5	21.5	33.5	38.5	55
Late Preterm Labor (34 36 6/7 w)	530	36.69	13.58	7.36	18.78	23.59	50.27	42.37
Late Normal pregnancy (34‐36 6/7 w)	110	24.57	7.29	9.14	20	39	31.86	59
Early Preterm Labor (20 0/7 33 6/7 w)	470	30.21	1.49	31.10	3.40	33.80	31.7	37.2
Early Normal pregnancy (20 0/7 33 6/7 w)	90	11.12	4.44	26.66	5.56	52.22	15.56	57.78

**FIGURE 4:**
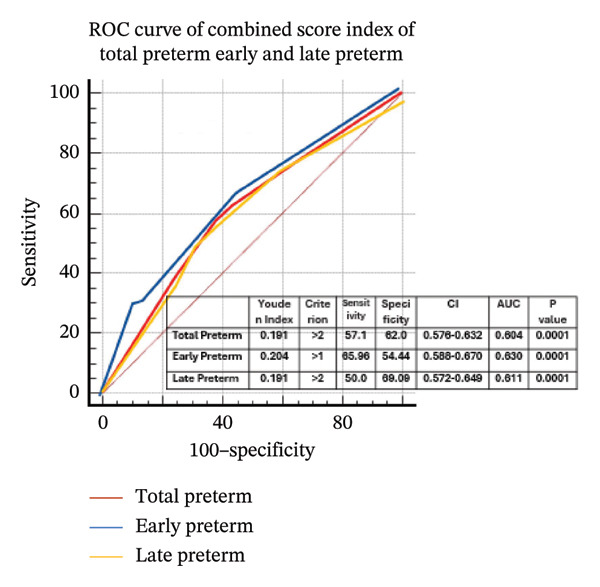
ROC curve of combined Score indices of neutrophils, platelets, NLR and PLR in total preterm, early (20 0/7–33 6/7 w) and late preterm (34–36 6/7 w).

The ROC curve used to evaluate the predictive values of the Combined Score Index of N, PLT count, NLR and PLR in the Total preterm, Early and Late preterm cases compared with normal pregnancy. AUC of the combined score index score was 0.604, 0.630, and 0.611 (Figure 4). Although AUC of combined indices was higher (*p* < 0.001), it clinically has a weak predictive value.

### 4.1. SII Results

**FIGURE 5:**
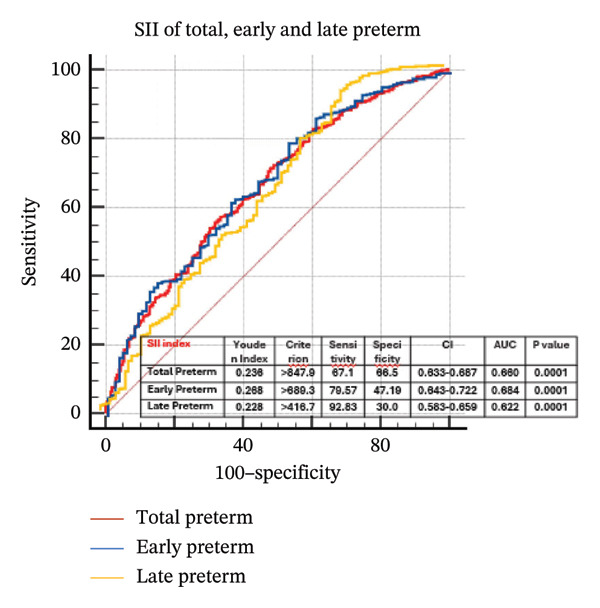
ROC curve of SII in total, early and late preterm labor.

AUC of SII in total, early, and late PTL was significantly higher compared with normal pregnancy (0.660, 0.684, and 0.622 and *p* 0.0001, 0.0001, and 0.0001) (Figure 5). SII had a weak clinical predictive value. Cutoff levels in total, early, and late PTL were 847.9, 689.3, and 416.7, respectively.

So, combined score index and SII although higher, clinically had a weak predictive value.

## 5. Discussion

PTL is a main cause of neonatal mortality and morbidity and has long‐term adverse effects on fetal health. The earlier the premature birth occurs, the greater the health risks will be. The prevention and prediction of preterm birth remain challenging issues [25].

The causes of preterm birth are multifactorial, and the precise etiology is unclear. Accumulating evidence suggests that inflammation (both clinical and subclinical) likely plays an essential role in triggering PTL [26]. However, subclinical intrauterine infection and inflammation are pathophysiology in over half of preterm cases. Sterile inflammation (inflammation without infection) has also been extensively reported in preterm birth [11–27].

Various studies have been devoted to identifying the potential risk factors for PTL. Inflammatory markers have been proposed as possible predictors of preterm deliveries. Additionally, a strong correlation between PTL and various inflammatory markers has been reported [28].

CBC and its derived parameters, including NLR and PLR, have recently been identified as inflammatory indicators for low‐grade inflammatory disorders [29, 30]. Many studies have used peripheral blood CBC as a simple, noninvasive, and cost‐effective test to predict spontaneous PTL. Recently, different inflammatory markers including CBC parameters have been recognized as predictive factors in various diseases [32, 33]. Since inflammation also occurs during childbirth, the function of inflammatory markers has been assessed in term and PTL [11–35].

### 5.1. Lymphocytes

During pregnancy, the lymphocyte counts decrease during the first and second trimesters and start to increase in the third trimester. Gezer et al. and Khatoon et al. found a decreased lymphocyte count in preterm groups [37, 38].

It was found that the lymphocyte count, at 24–34 gestational weeks, was significantly higher compared with normal pregnancy and could predict the time of birth [39]. On the other hand, many studies have reported no significant difference between the preterm and term groups regarding lymphocyte counts [15–40]. In our study, no significant changes in lymphocytes were observed between preterm and normal pregnancy.

### 5.2. N

It has been established that pregnancy is associated with mild neutrophilia. The increased N count occurs due to physiological stress of pregnancy or impaired N apoptosis [41]. As N play a major role in infection and inflammation, they are also liable to play a crucial role in preterm birth. The onset of term and PTL is usually accompanied by neutrophilia [42, 43]. The prediction of PTL using the peripheral blood N count has been studied in women with threatened PTL and PPROM [30–44].

Many researchers have stated that no significant variations were found in N count between PTL with or without PPROM [30–40].

On the other hand, Tolunay and Elci [39] found that the N count in threatened PTL at 24 weeks of gestational weeks was significantly higher compared with normal pregnancy and could predict the time of birth. Other researchers [37–47] have shown that patients delivered at LPL between 34 and 37 gestational weeks had significantly higher N levels.

Farzaneh et al. [48] reported that N were significantly higher in PTL but no significant alterations were found between EPL and LPL groups. Kurban et al. [47] found that N were higher in EPL compared with LPL.

In this study, N were significantly higher in PTL (Total, EPL, and LPL) (*p* = 0.0001, 0.0001, and 0.0003, respectively) compared with normal pregnancy. These findings were consistent with other studies by Yuan et al. [42]. Furthermore, our results are in line with the findings of Kurban et al. [47] where N in EPL were significantly higher compared with LPL (*p* = 0.0001) (Table 2).

### 5.3. NLR

NLR is an indirect marker of the host’s immune response [49]. It is a simple, cost‐effective, and dependable indicator of subclinical inflammation. Several studies have investigated the clinical usefulness of NLR during pregnancy (term, threatened preterm, early, and late preterm), chorioamnionitis, neonatal sepsis, and fetal birthweight [50].

NLR considerably increased in patients with labor at term approving that inflammatory mediators play a vital role in human parturition [51, 52]. Kim et al. [54] observed that NLR can be used as a predictor of placental inflammatory response (PIR) associated with PTL. Higher NLR was noted in those patients with the histologic evidence of chorioamnionitis. In premature labor, a NLR (> 6) was linked to a fivefold higher risk of histological chorioamnionitis. NLR has a useful predictive value and could be used as a diagnostic marker for histological chorioamnionitis in cases with preterm premature rupture of membranes before 34 weeks of gestation [47–55]. NLR was significantly elevated in PTL compared with those delivered at term [30–52]. These results are going with our results in total preterm compared to normal pregnancy (4.108 ± 1.889 and 3.384 ± 1.534, and *p* = 0.0001) (Table 1).

Kurban et al. [47] stated that the NLR value was 6.1 ± 3 in the EPL group, 4.5 ± 2.5 in the LPL patient group, and 3.9 ± 1.9 in the term patient group. Our results for NLR in this study were 4.41 ± 2.02, 3.84 ± 1.72, 3.56 ± 1.54, and 3.24 ± 1.52 in EPL, LPL, early, and late normal pregnancy, respectively.

Research has investigated the usefulness of NLR in predicting preterm delivery [61, 62]. In retrospective cohort studies [58–61], NLR was testified to be a risk factor for preterm delivery [58–62]. However, in other case–control studies, the researchers found that NLR was not a risk factor for preterm delivery [32–64].

Hyun‐Hwa Cha et al. [40] found that there were no significant differences in NLR between spontaneous PTL, PTL due to maternal, fetal, or placental causes and normal term deliveries. Additionally, Özdemir and Özdemir [65] reported that there was no significant difference in NLR between the preterm (EPL and LPL), early term, and late term groups. NLR was elevated in EPL than that in LPL [47].

These results are consistent with our own, which found that NLR rose in both EPL and LPL as compared with a normal pregnancy. EPL had considerably higher NLR levels than LPL (*p* = 0.0001).

### 5.4. PLTs in PTL

PLT counts tend to decline progressively during normal pregnancy as pregnancy progresses, but the levels remain within the normal range. On the 24th gestational week, this decline becomes statistically significant [6].

Patients with histological chorioamnionitis had a significantly greater PLT count than those in the control group [47]. Also, comparing the PPROM group (24–37 weeks) to the control group, the former had greater PLT counts [66]. In contrast, Urmila Kumari et al. [67] reported that the mean PLT count in women with PPROM between 24 and 36 weeks was lower than the mean PLT counts in women of normal pregnancy.

Many studies have shown that there is no significant difference in the mean values of PLTs between women with preterm, threatened preterm, normal pregnancy, or full‐term deliveries negating this marker as a good predictor of PTL [15–30].

However, in other studies, PLT levels were significantly higher in preterm cases compared with women delivered at term [48–64]. Rongli Xu et al. [70] showed that PLTs in preterm birth women were higher than those in full‐term and threatened preterm cases.

Our results showed that PLTs are significantly increased in total PTL compared with normal pregnancy (257.38 ± 71.134 and 231.88 ± 60.15, *p* = 0.0001) (Table 1 and Figure 1). Also, PLTs were significantly increased in EPL and LPL compared with the control groups (261.85 ± 71.94, 253.45 ± 70.18, 228.23 ± 54.35, and 234.83 ± 64.32, *p* < 0.0001 and 0.01, respectively) (Table 2). PLTs were also significantly higher in EPL compared with LPL (*p* < 0.05).

### 5.5. PLR in Preterm

High PLR is an important predictor of early pregnancy loss and threatened abortion [16]. In early premature rupture of membranes, PLR was significantly higher compared with normal pregnancy [66].

Numerous studies have found that PLRs are higher in PTL groups compared with normal pregnancy [48–50]. A recent study reported that PLR is robustly associated with spontaneous preterm birth [72]. Furthermore, mothers with higher PLT count and PLR also have increased risk for preterm delivery [35].

On the other hand, other investigators found no significant difference in PLR in threatened preterm (< 37 w), early preterm, and late preterm compared with term labor [30–32].

In this research, PLR was significantly higher in total PTL compared with normal pregnancy (115.49 ± 45.23 and 102.50 ± 42.37 and *p* = 0.0002) (Table 1).

PLR in EPL and LPL (118.38 ± 48.51, 112.92 ± 41.95, 100.58 ± 34.65, and 104.15 ± 47.65 and *p* = 0.0009 and 0.05, respectively) were higher compared with early and late normal pregnancy. Also, PLR was significantly higher in EPL compared with LPL (*p* = 0.05) (Table 2).

### 5.6. ROC Curve and Prediction of PTL

It is crucially important to predict and prevent preterm birth. Previous studies have shown that N count, NLR, PLT count, and PLR could predict PTL [73].

Many studies have utilized ROC analysis to assess the usefulness of these inflammatory markers in predicting preterm delivery. Typically, AUC, cutoff levels, sensitivity, and specificity were calculated [74, 75]. The AUC values for N count, NLR, PLT count and PLR were generally bad or weak productivity ranging between 0.5 and 0.7 in most previous studies with significant variations in cutoff levels [2–4]. A low or mild productivity of AUC suggests a high likelihood of both false positives (predicting disease when it is not present) and false negatives (not predicting disease when it is present). In our research, ROC curve analysis of total preterm and early and late preterm were weak (0.56–0.69) but statistically significant and not due to random chance (Figures 1, 2, and 3). This may suggest that they are most effective when combined with biophysical predictors (e.g., cervical length) to provide a more comprehensive risk profile for both EPL and LPL.

### 5.7. Combined Indices

Combining NLR and PLR into a composite index might harness the strengths of each and overcome their individual limitations, potentially improving overall predictive performance.

In general, combining markers can improve AUC, as a predictive model. In other fields like oncology and cardiology, combined inflammatory indices (NLR + PLR) have shown better prognostic performance than individual ratios.

Additive scoring systems (where all markers are equal and each marker contributes 0 or 1 point) are intuitive and easy for clinicians to calculate rapidly at the point of care without complex software, which we believe is a significant advantage for a potential screening tool for PTL.

### 5.8. Combined Score Index

A high score (3 + 4) was found in total, early, and late preterm labor (57%, 31.7%, and 50.27%) compared with normal pregnancy (38.5%, 15.56%, and 31.86%) (Table 3). This may reflect that PTL is correlated with a systemic inflammatory state involving N and PLT activation.

Combined Score Index was significantly higher in total, early, and late preterm compared with normal pregnancy (AUC 0.604, 0.630, and 0.611 and *p* 0.001, 0.001, and 0.001) with weak clinical productivity (Figure 4).

### 5.9. SII Discussion

Recently, several new inflammatory markers, such as SII, SIRI, and PIV, have been utilized to represent the inflammatory state. SII is an emerging, cost‐effective biomarker to assess the body’s inflammatory and immune status. In clinical research, it is being used for early predicting several pregnancy‐related conditions and complications such as gestational diabetes mellitus and preeclampsia [77]. The role of the SII in predicting preterm delivery and related complications has been investigated. It was reported by Özkan S, Yaprak Üstün [78], Gulsan Karabay et al. [79], and Hrubaru et al. [35] that no significant correlation was found between SII levels in preterm and normal delivery.

Kırat S [23] found a significantly higher SII of preterm than normal pregnancy (AUC: 0.599, cutoff: 783.48, sensitivity: 56.8%, and specifically: 57%). In cases of threatened preterm delivery, the SII has been revealed to have predictive value and can be used in clinical assessments [33]. Also, Sert ZS and Bülbül *R* [24] reported that SII was more effective than NLR and PLR in the early detection of PTL (AUC: 0.792 and cutoff 789.3, with 68.4% sensitivity and 81.5% specificity). SII can help detect pregnant women at risk of developing PTL [24].

In this study, the results of AUC of SII were significantly higher (AUC: 0.660, cutoff: 847.9, sensitivity: 67.1, and specifically: 66.5) (Figure 5). Although the ROC curve of Combined Score Index and SII are increased, they still have a weak predictive value (AUC: 0.604, 0.630, 0.611, 0.660, 0.684, and 0.622) in total, early, and late preterm labor.

## 6. Conclusion

Inflammatory markers are weak predictors, and clinicians may find limited utility in implementing these tests in routine practice for predicting PTL. A combination of multiple biomarkers may be more reliable for the prediction of PTL.

In recent years, some researchers have proposed N, NLR, PLTs, and PLR as new inflammation indicators to predict the occurrence of preterm birth. We investigated the diagnostic and predictive validities of some hematologic and inflammatory indices as potential risk factors for PTL. In conclusion, we found a significant increase in N, NLR, PLTs, and PLR in PTL (total, EPL, and LPT) compared with normal pregnancy. However, single parameters and combined indices have a weak clinical predictive value for prematurity.

These weak predictive values may be due to the involvement of other factors attributed to causing PTL such as genetic factors, placental problems, polyhydramnios, oligohydramnios, short cervix, irregularly shaped uterus, poor fetal growth, maternal age, smoking, overweight, underweight, high blood pressure, or diabetes. Factors such as nutrition, anemia, and socioeconomic conditions should be also considered.

The increase in N, NLR, PLT, and PLR in preterm birth may also be related to physiological changes during pregnancy, maternal conditions such as infections (urinary tract infection and respiratory infection), hypertensive disorders, anemia, obesity, and others. The stress of PTL may play a role in these CBC changes. Presence or absence of PPROM and timing of collecting data before or after triggering labor may also play a role in the changes in results in different studies.

In comparing predictive values of N, PLTs, NLR, and PLR, combined score system and SII, it is clear that no single method is perfect. No single biomarker has yet been proved to fulfill all the requirements of a clinically dependable predictive test for spontaneous preterm birth. The inflammatory markers associated with PTL may be insufficient as a standalone screening tool without additional clinical parameters. More research is needed to find suitable markers for preterm predictivity. Also, combining inflammatory markers with other markers such as cervical length, fetal fibronectin, and proteomics will improve the predictive values of PTL.

### 6.1. Strengths

The topic is highly relevant, addressing a critical issue in obstetrics where PTL affects a significant percentage of pregnancies and has serious implications for neonatal health. The study includes a substantial sample size of 1000 patients, which enhances the reliability of the findings.

### 6.2. Limitation

This study involved a single center. Multiple centers are needed to improve the validity of the findings. Due to the study’s retrospective design, biases in patient selection and data collection may be introduced. Incomplete data or incorrect case classification might result from retrospective studies, especially in complex conditions like premature labor. Accurate gestational age remains essential for diagnosis of neonatal immaturity but does not define underlying disease mechanisms. Careful ultrasound, Doppler, placental function markers, fetal abnormalities, congenital anomalies, cervical length, cervical dilatation, maternal immune response, and infections should be investigated [80]. Addressing these issues in future research could enhance their impact on clinical practice. Combined Score Index currently lacks external validation in an independent cohort. Future studies involving multicenter, prospective cohorts are required to confirm these cutoff values and assess the scoring system’s generalizability across different populations.

**Nomenclature**
N: Neutrophils
PLTs: Platelets
NLR: Neutrophil–lymphocyte ratio
PLR: Platelet–lymphocyte ratio
LPL: Late preterm labor
EPL: Early preterm labor
AUC: Area under the receiver operating characteristic curve
ROC: Receiver operating characteristics.

## Author Contributions

Dr. Emad Elmaradny was responsible for conceptualization, supervision, formal analysis, writing–review and editing: developing research questions and hypotheses, guiding teams in refining methods, and interpreting results. Performing statistical tests to compare different groups within a study or evaluate Changes. Providing review input of figures, tables, and Supporting Information. Dr. Saud Aman was responsible for data curation: developing or implementing data preservation strategies. Dr. Mohammed Alaskar was responsible for methodology: determining study design such as participant selection, materials, settings, data characteristics, data collection, measurement, and analysis techniques. Dr. Reem Alnefayi was responsible for investigation: testing research hypotheses and documenting the research process. Searching and reviewing literature, samples, data, and other evidence. Dr. Ali Almuhesseny was responsible for project administration: managing correspondence with team members. Dr. Hashem Alshreef was responsible for resources: inventory management, safekeeping of samples, and providing reports on availability and state of resources and computing resources or other analysis tools. Dr. Malak Alshammari was responsible for methodology: materials, settings, data characteristics, data collection, measurement, and analysis techniques. Dr. Adnan Alhazmi was responsible for visualization: using data to create charts, graphs, or figures. Dr. Lulwah Altraifi was responsible for methodology: determining study design such as participant selection, materials, settings, data characteristics, data collection, measurement, and analysis technique. Dr. Amal AlSaedi was responsible for writing–original draft: creating the first and full version of an article.

## Funding

No funding was received for this study. None of the authors have any financial or personal relationships with other people or organizations that could inappropriately influence or bias their work.

## Ethics Statement

The Ethical Committee of Riyadh Health Second Cluster approved the study, and the Institutional Review Board (IRB) was obtained.

## Conflicts of Interest

The authors declare no conflicts of interest.

## Data Availability

The data are not publicly available due to privacy restrictions. Personal data such as name address phone number and ID were blind to researchers. Data used to support the findings of this study are available on reasonable request from the corresponding author.
